# Safety and effectiveness of baloxavir marboxil and oseltamivir for influenza in children: a real-world retrospective study in China

**DOI:** 10.3389/fped.2024.1418321

**Published:** 2024-07-29

**Authors:** Xilin Ge, Yang Chen, Wei Wu, Jinmiao Lu, Yi Wang, Zhiping Li

**Affiliations:** ^1^ Department of Pharmacy, National Children's Medical Center, Children's Hospital of Fudan University, Shanghai, China; ^2^ Department of Neurology, National Children's Medical Center, Children's Hospital of Fudan University, Shanghai, China

**Keywords:** baloxavir marboxil, oseltamivir, safety, effectiveness, child

## Abstract

**Objectives:**

To evaluate the safety and effectiveness of baloxavir marboxil (baloxavir) and oseltamivir in pediatric influenza patients in China.

**Methods:**

Patients filling a prescription for baloxavir or oseltamivir within 48 h following an influenza-related outpatient visit were identified in Children's Hospital of Fudan University in China between March 2023 and December 2023. Outcomes were assessed after antiviral treatment and included the incidence of adverse reactions and the duration of fever and other flu symptoms.

**Results:**

A total of 1430 patients infected with influenza A were collected and 865 patients (baloxavir: *n* = 420; oseltamivir: *n* = 445) finally included. The incidence of adverse reactions of nausea and vomiting was significantly different between the baloxavir group (2.38%) and the oseltamivir group (12.13%) [*P *< 0.001, OR = 4.2526, 95%CI (2.0549, 9.6080)]. No differences in other adverse reactions were observed between the two groups. The mean duration of fever in baloxavir group (1.43d) was significantly shorter than that in oseltamivir group (2.31d) [*P *< 0.001, 95%CI (0.7815, 0.9917)]. There were no differences in the mean duration of nasal congestion and runny nose, sore throat, cough, and muscle soreness between two groups.

**Conclusions:**

The incidence of nausea and vomiting is lower with baloxavir compared to oseltamivir, and the duration for complete fever reduction is shorter with baloxavir than with oseltamivir. The results indicate that baloxavir is well tolerated and effective in Chinese children.

## 1 Introduction

Influenza, a respiratory illness of varying severity, annually infects an estimated 3%–11% of individuals in the United States, placing a substantial burden on the healthcare system (1). The US Centers for Disease Control and Prevention has estimated that influenza has resulted in an annual average of 9.3 million to 41 million illnesses, 100,000–710,000 hospitalizations, and 4,900–51,000 deaths during the period from 2010 to 2023 (2). Despite the fact that most individuals with influenza will experience recovery within a two-week timeframe, the infection has the potential to result in moderate complications like sinus and ear infections, as well as severe complications such as pneumonia, myocarditis, encephalitis, myositis, and multiple organ failure. Furthermore, influenza can exacerbate pre-existing chronic conditions such as asthma, chronic obstructive pulmonary disease, and heart disease (3). The inflammation in the respiratory system caused by the influenza virus can harm tissues and lung function, potentially leading to lower respiratory tract infections and other respiratory illnesses (4). Individuals at higher risk of severe influenza-related complications comprise adults aged over 65, residents of long-term care facilities, individuals with specific chronic conditions, and children under 2 years old (5, 6). Influenza infects individuals across all age groups, with a notably high incidence rate among children. Among these, the youngest children are particularly susceptible to virus-induced damage, secondary bacterial infections, and associated complications (7, 8). The variation in child mortality is tied to the seasons, with factors such as virus subtype, pre-existing immunity, and underlying diseases playing a significant role (9, 10). Recent estimates suggest that 92 countries report between 9,000 and 106,000 influenza-related deaths annually, predominantly affecting children under the age of 5, with a median figure of 4,888. In the United States, the overall cumulative mortality rate for children with influenza is 0.15 deaths per 100,000 children. This rate is notably higher among infants under six months, with a cumulative mortality rate of 0.66 deaths per 100,000 children (11). Children significantly contribute to influenza dissemination in the community as they are highly susceptible to infection, exhibit elevated incidence rates, have prolonged viral shedding, and engage in frequent contact with others in their families and communities (12, 13).

Antiviral therapy serves as a valuable complement to influenza immunization in symptom management and the prevention of secondary complications, such as bacterial infections (14). According to expert guidelines, antiviral therapy should be initiated within 48 h for patients experiencing more severe illness or those at higher risk of complications (15). The current standard of care involves promptly initiating neuraminidase inhibitors (e.g., oseltamivir) for influenza treatment, which effectively alleviates symptoms, reduces complications, minimizes healthcare resource utilization, and lowers mortality rates in hospitalized patients (16–21).

Baloxavir marboxil (hereafter as “baloxavir”) received FDA approval in October 2018 as an oral single-dose treatment for influenza. Distinguished from neuraminidase inhibitors, baloxavir functions as a cap-dependent endonuclease inhibitor, impeding viral RNA transcription and halting viral replication (22). In a phase III clinical trial conducted among outpatients presenting with influenza-like illness, baloxavir exhibited a significant decrease in the time required for alleviation of influenza symptoms in comparison to placebo (53.7 h vs. 80.2 h; *P *< 0.001), showing equivalence in effectiveness to a 5-day regimen of twice-daily oseltamivir (23). In a separate phase III trial conducted on outpatients with a high susceptibility to influenza complications, predominantly individuals with asthma, chronic lung disease, and endocrine disorders, including diabetes, treatment with baloxavir resulted in a notably swifter resolution of influenza symptoms in contrast to placebo (73.2 h vs. 102.3 h; *P *< 0.001) and a marginally quicker resolution compared to oseltamivir (81.0 h; *P *= 0.8347) (24). In high-risk patients, treatment with baloxavir led to a notable decrease in complications as opposed to placebo treatment (2.8% vs. 10.4%; *P *< 0.001), with the reduction in sinusitis and bronchitis serving as the main factors contributing to this disparity (24).

Despite previous research showing the efficacy of baloxavir and oseltamivir in treating influenza, it is still uncertain whether there are distinct clinical variations in the safety and effectiveness of these two drugs in children with influenza in China. The current lack of clarity emphasizes the requirement for more investigations and evaluations to completely comprehend the possible benefits and risks of these antiviral medications for pediatric patients in China. In this retrospective study, we analyzed Chinese children who received either baloxavir or oseltamivir for influenza treatment. The analysis involved a comparison of adverse reactions and treatment outcomes between the two drugs, along with an assessment of their safety and effectiveness.

## 2 Methods

### 2.1 Study design

The study was a randomized retrospective control cohort trial that enrolled outpatients 0–18 years of age with influenza A in Children's Hospital of Fudan university in China from March 2023 to December 2023. Through the medical record system search, it is determined that patients aged 0–18 who were prescribed baloxavir or oseltamivir are required to have continuous registration data at least 6 months before and 1 month after the index date of antiviral prescriptions. The prescription must be issued within 2 days after the flu-related outpatient service. For any patient, the study only included the first influenza-related outpatient visit, and then received the relevant antiviral prescription within 2 days. In addition, patients cannot receive control antiviral drugs within one month of the index prescription, nor can they take any other antiviral drugs as supplements. And all the patients’ information was collected and collated through medical record retrieval and telephone survey.

### 2.2 Patients

Patients who were enrolled had fever (axillary temperature, ≥38.0℃), and a rapid detection of antigen showed positive for influenza. For the study population, the inclusion criteria are as follows: (1) The age of patients is 0–18 years old, regardless of sex; (2) The clinical test results are diagnosed as influenza infection; (3) Administer baloxavir or oseltamivir for treatment and complete the course of treatment according to the doctor's advice. Exclusion criteria are: (1) Refusal of informed consent or loss to follow up; (2) Using two or more drugs studied at the same time or taking other antiviral drugs in combination to ensure that the results of this study are attributed to a single drug; (3) Administer medicine >2 days (>48 h) after onset of symptoms. Since the typical duration of influenza is about 5–10 days, we excluded cases where medication is administered too late, which may be essential to avoid the impact of natural recovery when evaluating the effectiveness of drug treatments.; (4) Combined with other complicated diseases. In addition, baloxavir is only suitable for influenza treatment in children over 5 years old in China, and patients under 5 years were given informed consent and dosage adjustment.

### 2.3 Dosage and administration

Patients were administered a single oral dose of baloxavir on day 1 and a regimen of twice a day oral dose for five days of oseltamivir from day 1 to day 5. Dosage of two drugs should be in accordance with the instructions. For baloxavir, children ≥5 years received 40 mg baloxavir if their body weight was <80 kg and 80 mg baloxavir if their body weight was ≥80 kg. In China, baloxavir can only be approved for children over 5 years old and adults with influenza, so children <5 years received 1 mg/kg as an adjustment dose after informed consent. And for oseltamivir, children ≥13 years received 75 mg oseltamivir twice a day. Children <13 years received 30 mg oseltamivir twice a day if their body weight was ≤15 kg, 45 mg oseltamivir twice a day if their body weight was 15–23 kg, and 60 mg oseltamivir twice a day if their body weight was 23–40 kg, and 75 mg oseltamivir twice a day if their body weight was >40 kg. Children who did not complete the regimen of treatment correctly according to the doctor's instruction were excluded.

### 2.4 Patient demographics and baseline characteristics

Demographic data included patient age, sex, weight, and the date of treatment. Baseline clinical data included time from symptom onset to treatment, medication prescription, peak body temperature, past history, liver and kidney function.

### 2.5 Outcomes

Cumulative outcomes were assessed within 30 days after index prescription fill (excluding date of prescription). The end point of safety assessment was the incidence of adverse drug reactions(ADRs), including dizziness, headache, nausea and vomiting, diarrhea, rash and mental symptoms. The end point of effectiveness assessment was the duration of influenza-infected symptoms, such as fever, nasal congestion and runny nose, sore throat, cough and muscle soreness.

### 2.6 Statistical analysis

The cohorts were compared with the Chi-squared test and Fisher's exact test for categorical measures. A *p*-value less than 0.05 indicates a statistically significant difference between the groups. Propensity score matching (PSM) was conducted between the two groups. A random forest algorithm was used to estimate the propensity scores which were calculated based on sex, age and weight. One-to-one nearest neighbor matching without replacement was performed and the standardized mean differences of less than 0.13 for each covariate was considered indicative of a good balance. Analyses were conducted using IBM SPSS statistics version 25 and R 4.3.2. Furthermore, a logistic regression analysis was performed to examine the incidence of nausea and vomiting adverse reactions, while a linear regression analysis was conducted to assess the duration of fever between the two groups.

## 3 Results

### 3.1 Analysis populations

**Figure 1:**
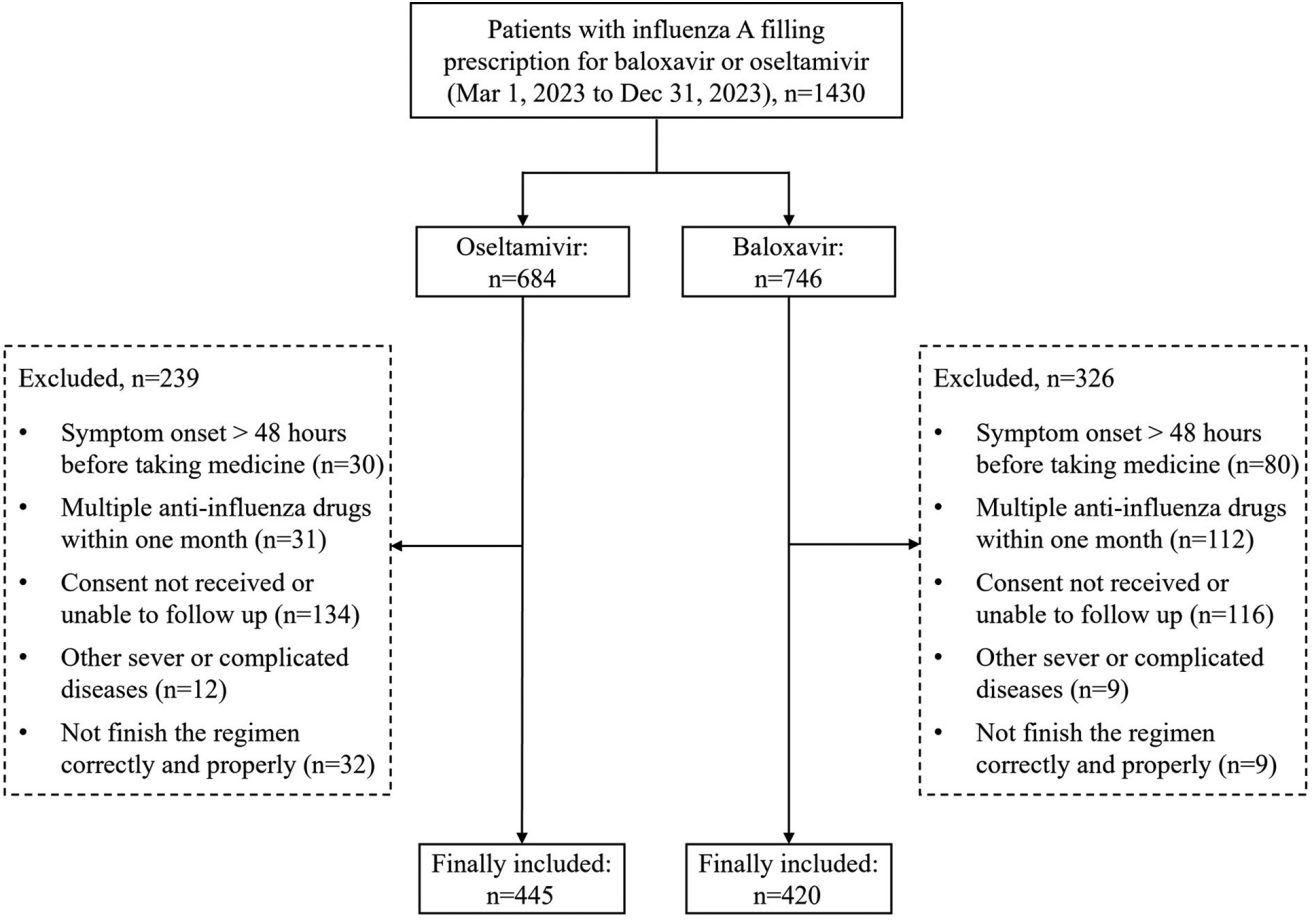
Patient disposition.

A total of 1,430 influenza A-infected-patients who filled a prescription for baloxavir or oseltamivir during March to December in 2,023 were collected. Through exclusion and screening, 865 patients (baloxavir: *n* = 420; oseltamivir: *n* = 445) were finally included. 239 patients were excluded in oseltamivir group because of a failure to satisfy the major criteria, and 326 in baloxavir group. The main reasons for exclusion included symptom onset >48 h before taking medicine (30 in oseltamivir group, 80 in baloxavir group), multiple anti-influenza drugs within one month (31 in oseltamivir group, 112 in baloxavir group), consent not received or unable to follow up (134 in oseltamivir group, 116 in baloxavir group), other sever or complicated diseases (12 in oseltamivir group, 9 in baloxavir group), and not finish the regimen correctly and properly (32 in oseltamivir group, 9 in baloxavir group) (Figure 1).

Of 445 patients in oseltamivir group, most patients were 5–12 years old and the age range from 0.5 to 17.2 years with a median age of 7.3 years. Approximately 3.37% of patients were aged <2 years, approximately 16.18% were 2–5 years, approximately 72.13% were 5–12 years and approximately 8.32% were ≥12 years. More patients were male than female (54.61% vs. 45.39%). The mean weight was 29.62 kg. And of 420 patients in baloxavir group, the median age was 11.5 years and no one was under 2 years old. Approximately 3.10% of patients were 2–5 years, 53.33% were 5–12 years and 43.57% were ≥12 years. 60.95% of the patients were male and the mean weight was 44.40 kg. In two groups, the median time until treatment (24 h in oseltamivir group vs. 28 h in baloxavir group) and the peak body temperature before treatment (39.3℃ in oseltamivir group vs. 39.2℃ in baloxavir group) were both close. Besides, we performed propensity score matching (PSM) on the two groups of patients to ensure balanced baseline data. After matching, a total of 492 patients were included (oseltamivir: *n* = 246, baloxavir: *n* = 246), with no statistically significant differences in baseline characteristics between the two groups (Table 1).

**Table 1:** Baseline characteristics of finally included patients.

Characteristic	Unmatched			Matched		
	Oseltamivir (*n* = 445)	Baloxavir (*n* = 420)	*P*	Oseltamivir (*n* = 246)	Baloxavir (*n* = 246)	*P*
Age, median (range), y	7.3 (0.5–17.2)	11.5 (2.8–17.3)	<0.001	9.1 (1.1–17.2)	9.9 (2.8–16.1)	0.106
Age groups in years, *n* (%)			<0.001			0.304
<2	15 (3.37)	0 (0)		3 (1.22)	0 (0)	
2–5	72 (16.18)	13 (3.10)		18 (7.32)	13 (5.28)	
5–12	321 (72.13)	224 (53.33)		187 (76.02)	192 (78.05)	
≥12	37 (8.32)	183 (43.57)		38 (15.45)	41 (16.67)	
Sex, *n* (%)			0.059			0.587
Male	243 (54.61)	256 (60.95)		131 (53.25)	137 (55.69)	
Female	202 (45.39)	164 (39.05)		115 (46.75)	109 (44.31)	
Weight, mean (SD), kg	29.62 (10.43)	44.40 (14.85)	<0.001	34.72 (9.65)	35.63 (10.44)	0.315
Time until treatment, median(range), h	24 (7–46)	28 (5–48)	0.632	24 (7–46)	27 (5–48)	0.675
Peak body temperature, mean(SD), °C	39.3 (0.75)	39.2 (0.68)	0.332	39.3 (0.73)	39.4 (0.71)	0.731

### 3.2 Safety analysis

**Figure 2:**
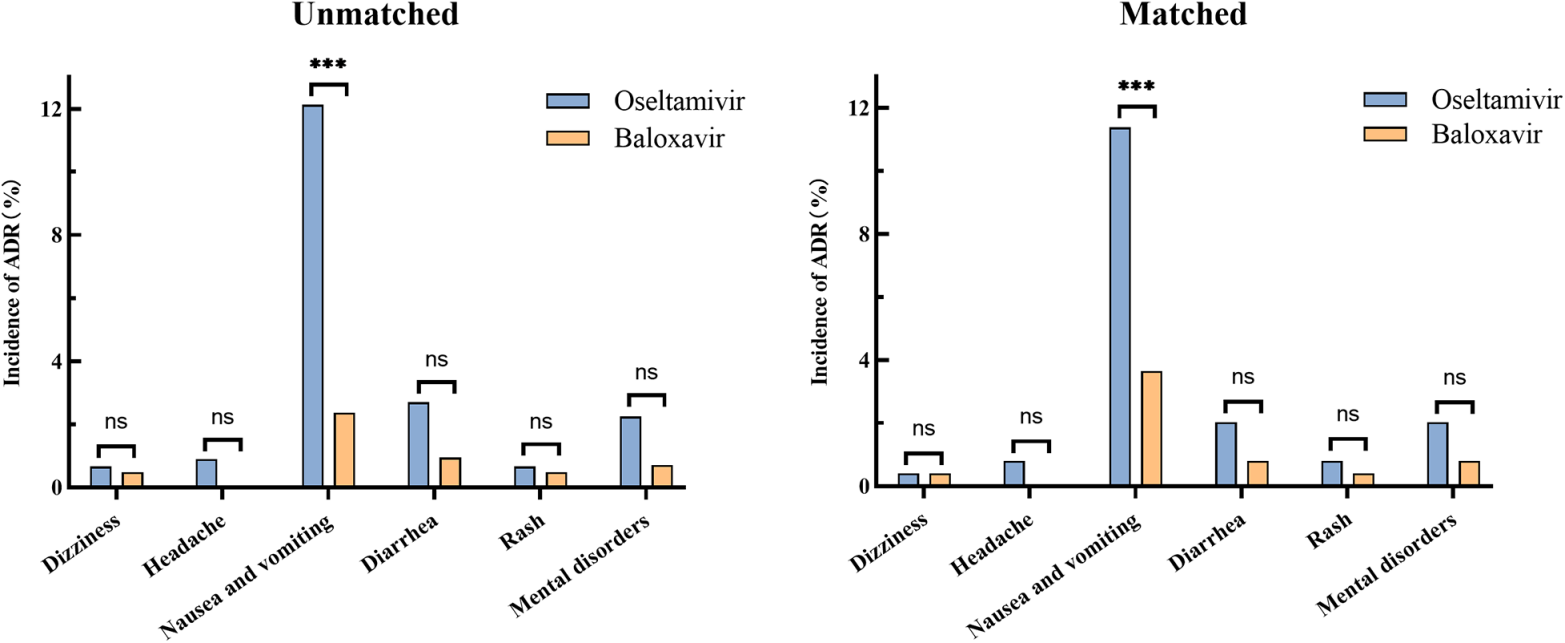
Incidence of adverse reactions in oseltamivir and baloxavir groups before and after propensity score matching. The statistical significance was assessed using the Chi-squared test, providing *p*-values to determine the independence between the groups. (ns, no significant difference; ***P < 0.001).

Before matching, adverse drug reactions occurred in 21 (5.00%) of 420 patients who received baloxavir and in 86 (19.30%) of 445 patients who received oseltamivir, which showed a significant difference. Serious adverse events were not noted in both baloxavir group and oseltamivir group. In oseltamivir group, all reported ADRs were nausea and vomiting (12.13%), diarrhea (2.70%), mental disorders (2.25%), headache (0.90%), rash (0.67%) and dizziness (0.67%). And in baloxavir group, the ADRs were nausea and vomiting (2.38%), diarrhea (0.95%), mental disorders (0.71%), dizziness (0.48%) and rash (0.48%). The most common ADR in both groups was nausea and vomiting with a significantly higher incidence in oseltamivir group than in baloxavir group (*P *< 0.001). There were no significant differences in other ADRs between the two groups (Figure 2). After matching, adverse drug reactions were observed in 43 out of 246 patients (17.48%) who received oseltamivir, compared to 15 out of 246 patients (6.10%) who received baloxavir, demonstrating a significant difference. The conclusions regarding adverse drug reactions remained consistent both prior to and following the matching process, as detailed in the accompanying table (Table 2). Besides, our analysis of the influence of sex on the incidence of adverse reactions in two groups revealed no significant difference between male and female patients (Table 3).

**Table 2:** Adverse drug reactions in oseltamivir and baloxavir groups.

Adverse drug reaction	Unmatched			Matched		
	Oseltamivir (*n* = 445)	Baloxavir (*n* = 420)	*P*	Oseltamivir (*n* = 246)	Baloxavir (*n* = 246)	*P*
Total, *n* (%)	86 (19.30)	21 (5.00)	<0.001	43 (17.48)	15 (6.10)	<0.001
Dizziness, *n* (%)	3 (0.67)	2 (0.48)	1.000	1 (0.41)	1 (0.41)	1.000
Headache, *n* (%)	4 (0.90)	0 (0)	0.148	2 (0.81)	0 (0)	0.499
Nausea and vomiting, *n* (%)	54 (12.13)	10 (2.38)	<0.001	28 (11.38)	9 (3.66)	0.001
Diarrhea, *n* (%)	12 (2.70)	4 (0.95)	0.057	5 (2.03)	2 (0.81)	0.450
Rash, *n* (%)	3 (0.67)	2 (0.48)	1.000	2 (0.81)	1 (0.41)	1.000
Mental disorders, *n* (%)	10 (2.25)	3 (0.71)	0.064	5 (2.03)	2 (0.81)	0.450

**Table 3:** Adverse drug reactions of oseltamivir and baloxavir by sex.

Adverse drug reaction	Oseltamivir			Baloxavir		
	Male (*n* = 243)	Female (*n* = 202)	*P*	Male (*n* = 256)	Female (*n* = 164)	*P*
Dizziness, *n* (%)	2 (0.8)	1 (0.5)	1.000	2 (0.8)	0 (0)	0.523
Headache, *n* (%)	1 (0.4)	3 (1.5)	0.234	0 (0)	0 (0)	/
Nausea and vomiting, *n* (%)	36 (14.8)	18 (8.9)	0.058	5 (2.0)	5 (3.0)	0.472
Diarrhea, *n* (%)	5 (2.1)	7 (3.5)	0.361	1 (0.4)	3 (1.8)	0.139
Rash, *n* (%)	2 (0.8)	1 (0.5)	1.000	0 (0)	2 (1.2)	0.152
Mental disorders, *n* (%)	5 (2.1)	5 (2.5)	0.767	3 (1.2)	0 (0)	0.284

The logistic regression analysis also revealed a significant effect of the two drugs on the incidence of vomiting. The results were statistically significant, with a *p*-value of less than 0.001, indicating a strong association between the drugs and the incidence of vomiting. The odds ratio (OR) was calculated to be 4.2526, suggesting that the likelihood of vomiting was over four times higher in the oseltamivir group compared to the baloxavir group. The 95% confidence interval for the odds ratio ranged from 2.0549 to 9.6080, further confirming the robustness of these findings. Other factors, including age, weight, sex, time until treatment and peak body temperature, did not show a significant difference in the incidence of adverse reactions between the two groups (Table 4). The results indicated that these variables did not contribute meaningfully to the variation in adverse reaction rates, suggesting that the observed differences in vomiting incidence were primarily attributable to the drug treatments rather than demographic or physiological characteristics.

**Table 4:** Results of logistic regression analysis on the incidence of adverse vomiting reactions.

Variable	Coefficient	Std. error	Z	*P*	OR	95% CI
Intercept	−6.53	7.195	−0.908	0.364	0.0015	(9.20e−10, 1.73e+3)
Drug (oseltamivir)	1.45	0.390	3.711	<0.001	4.2526	(2.0549, 9.6080)
Age	−0.02	0.089	−0.230	0.818	0.9797	(0.8248, 1.1665)
Weight	−0.01	0.024	−0.566	0.571	0.9862	(0.9389, 1.0324)
Sex	−0.41	0.279	−1.474	0.140	0.6626	(0.3783, 1.1357)
Time until treatment	−0.29	0.316	−0.914	0.361	0.7487	(0.3910, 1.3593)
Peak body temperature	0.11	0.183	0.627	0.530	1.1219	(0.7850, 1.6132)

It is important to note that besides nausea and vomiting, the incidence of ADRs of diarrhea and mental disorders in the two groups was also high, and especially the mental disorders needed more attention. We observed that in patients experiencing adverse drug reactions involving mental disorders, the administration of oseltamivir or baloxavir was linked to symptoms including agitation accompanied by shouting, incoherent speech, loss of consciousness during sleep, and subsequent anterograde amnesia. Although all the ADRs collected in the study were non-serious, we still classified the mental disorders as serious adverse reactions worthy of attention, in view of their physical damage to children and possible unknown sequelae.

### 3.3 Effectiveness analysis

**Figure 3:**
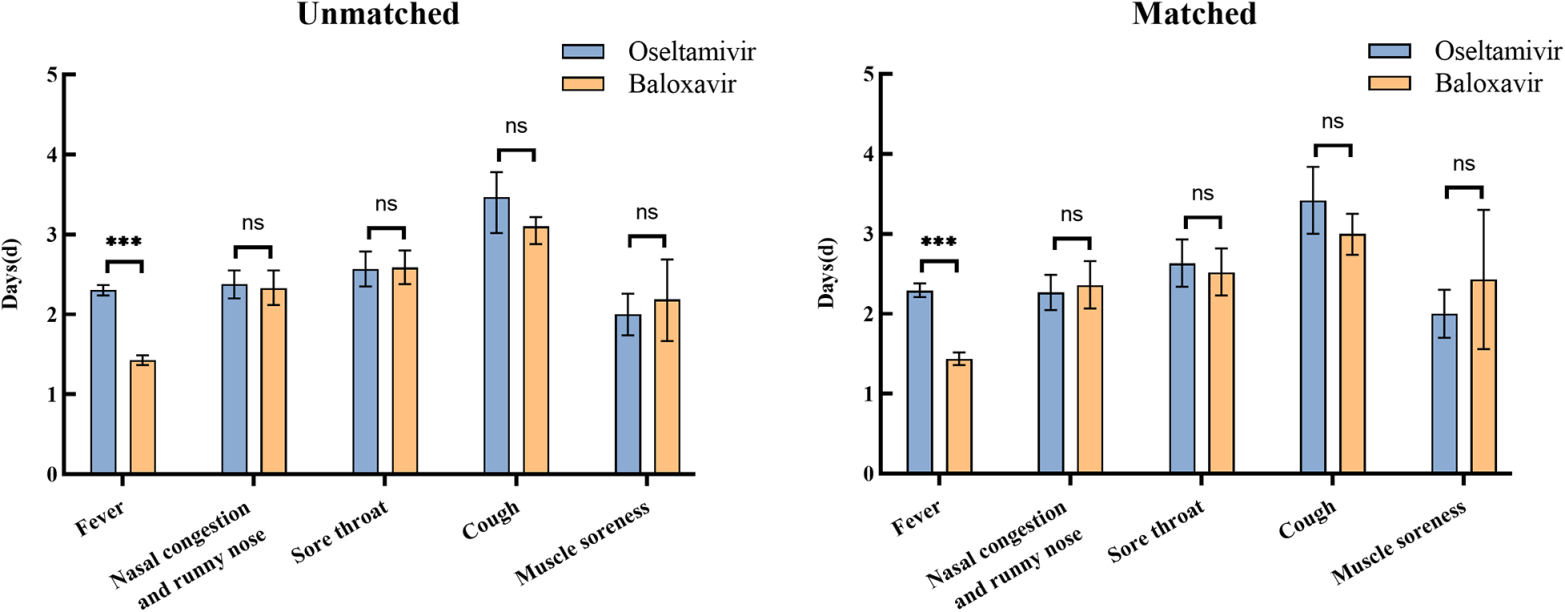
Duration of symptoms in oseltamivir and baloxavir groups before and after propensity score matching. The statistical significance was assessed using Fisher's exact test for group independence with corresponding *p*-values. (ns, no significant difference; ***P < 0.001).

The duration of symptoms was analyzed in baloxavir group and oseltamivir group before and after matching, and the same results were obtained. The mean [95% confidence interval (CI)] time to resolution of fever was 2.31 (2.24–2.37) days in oseltamivir group, while the mean time to resolution of fever in baloxavir group was 1.43 (1.37–1.49) days, which differed significantly between the two groups (*P* < 0.001). Other symptoms included nasal congestion and runny nose (112 in oseltamivir group and 182 in baloxavir group), sore throat (100 in oseltamivir group and 180 in baloxavir group), cough (102 in oseltamivir group and 271 in baloxavir group), and muscle soreness (35 in oseltamivir group and 43 in baloxavir group). There were no differences in the mean duration of the above symptoms between baloxavir and oseltamivir groups (Figure 3). Post-matching analysis revealed that only the mean time to fever resolution showed a significant difference between the two groups (*P* < 0.001). The oseltamivir group had a mean [95% confidence interval (CI)] fever resolution time of 2.29 (2.21–2.38) days, whereas the baloxavir group had a mean time of 1.44 (1.36–1.52) days. No significant differences were observed between the groups regarding the resolution times of other symptoms (Table 5).

**Table 5:** Duration of symptoms in oseltamivir and baloxavir groups.

Symptoms	Unmatched			Matched		
	Duration of symptoms, mean (95% CI), days			Duration of symptoms, mean (95% CI), days		
	Oseltamivir	Baloxavir	*P*	Oseltamivir	Baloxavir	*P*
Fever	2.31 (2.24, 2.37), *n* = 445	1.43 (1.37, 1.49), *n* = 420	<0.001	2.29 (2.21, 2.38), *n* = 246	1.44 (1.36, 1.52), *n* = 246	<0.001
Nasal congestion and runny nose	2.38 (2.20, 2.55), *n* = 112	2.33 (2.12, 2.55), *n* = 182	0.784	2.27 (2.05, 2.49), *n* = 66	2.36 (2.07, 2.66), *n* = 106	0.665
Sore throat	2.57 (2.35, 2.79), *n* = 100	2.59 (2.38, 2.80), *n* = 180	0.894	2.63 (2.34, 2.93), *n* = 60	2.52 (2.23, 2.82), *n* = 102	0.626
Cough	3.47 (3.02, 3.78), *n* = 102	3.10 (2.88, 3.22), *n* = 271	0.071	3.42 (3.00, 3.84), *n* = 57	3.00 (2.74, 3.25), *n* = 166	0.094
Muscle soreness	2.00 (1.74, 2.26), *n* = 35	2.19 (1.67, 2.69), *n* = 43	0.535	2.00 (1.70, 2.30), *n* = 26	2.43 (1.56, 3.31), *n* = 23	0.312

Similar results were observed in the linear regression analysis, which demonstrated a significant effect of the two drugs on the duration of fever. The analysis yielded statistically significant results (*P* < 0.001), with a 95% confidence interval ranging from 0.7815 to 0.9917. These consistent findings indicated that the mean time to fever resolution following baloxavir administration is shorter than that observed with oseltamivir. The results may suggest a potential superior efficacy of baloxavir in reducing fever duration compared to oseltamivir. Conversely, other variables such as age, weight, sex, time until treatment, and peak body temperature did not exhibit a significant impact on the duration of fever, suggesting that these factors did not influence the treatment outcome (Table 6). This highlights the robustness of the observed drug effect, independent of demographic and clinical characteristics.

**Table 6:** Results of linear regression analysis on the duration of fever.

Variable	Coefficient	Std. error	*t*	*P*	95% CI
Intercept	0.668	1.267	0.527	0.598	(−1.8190, 3.1560)
Drug	0.887	0.054	16.552	<0.001	(0.7815, 0.9917)
Age	−0.007	0.013	−0.555	0.579	(−0.0315, 0.0176)
Weight	0.003	0.003	1.074	0.283	(−0.0026, 0.0089)
Sex	0.026	0.047	0.548	0.584	(−0.0662, 0.1175)
Time until treatment	−0.058	0.050	−1.169	0.243	(−0.1564, 0.0396)
Peak body temperature	0.019	0.032	0.578	0.563	(−0.0446, 0.0818)

## 4 Discussion

This randomized retrospective control cohort trial assessed the incidence rate of ADRs and the duration of symptoms in patients infected with influenza A aged <18 years who were treated with either baloxavir or oseltamivir in China. Baloxavir was well tolerated, with no notable difference in safety compared with previous studies (23, 25). In Chinese children, baloxavir demonstrated great safety with a low incidence of non-serious adverse drug reactions (ADRs), primarily nausea and vomiting. Although the observed ADRs were mild, mental disorders associated with oseltamivir and baloxavir warrant attention. Symptoms such as delirium, hallucinations, and nocturnal screaming can cause significant concern among parents and potentially lead to physical and mental harm. Children are more susceptible to febrile delirium and convulsions than adults (23, 26, 27). Therefore, children infected with influenza should receive special attention and care when taking oseltamivir or baloxavir.

As with the safety results, the effectiveness of baloxavir for the treatment of influenza was similar to that seen in previous trials (23–25). The resolution of fever after baloxavir treatment was notably faster compared to oseltamivir, although the time to symptom alleviation was similar between the two groups. Previous studies comparing the duration of fever and symptoms between the baloxavir and oseltamivir treatment groups for influenza infected children did not show any difference. Specifically, in a randomized controlled study for children 1–12 years old, the median duration of fever was 41.2 vs. 46.8 h, and that of symptoms was 66.4 vs. 67.9 h for influenza A infections treated with baloxavir or oseltamivir, respectively (28). Likewise, another observational study conducted in Japan during the 2018–2019 season reported that the fever duration following baloxavir treatment did not differ from that with oseltamivir in influenza A infected children (25). However, a study involving both adolescents and adults demonstrated results consistent with our findings. This investigation assessed the efficacy of oseltamivir and baloxavir in treating influenza A infections among Chinese adolescents and adults. It revealed that the duration of fever was significantly shorter in the baloxavir group, averaging 1.5 (1.0–2.5) days, compared to the oseltamivir group, which averaged 2.5 (1.5–3.0) days, with a *P*-value of less than 0.001 (29). Our research findings diverged from the aforementioned studies regarding the duration of fever reduction in children. The potential reasons for this disparity are as follows. To begin with, there exist ethnic and racial variations. This study focused on children in China, whereas the previously mentioned research groups comprised Japanese or European participants. This difference in demographic may lead to slight variations in the findings of these studies, although this remains speculative and conjectural. Secondly, there exists a variance in drug resistance. This study was conducted right after the approval of baloxavir for treating influenza in children aged 5–12 years. Until then, baloxavir was exclusively prescribed for treating influenza in individuals aged 12 and older in China. Compared to Japan, where widespread use of baloxavir has resulted in a certain level of population resistance (28, 30–32), the Chinese population in this study exhibits lower resistance to baloxavir. As a result, the therapeutic effect may be more substantial, resulting in slightly divergent research outcomes. Nonetheless, this is merely a conjecture. Currently, there is a lack of knowledge regarding the prevalence of baloxavir resistance among children in China, and it may require assessment and validation through future drug resistance studies. Furthermore, the effects of antipyretic and analgesic drugs need to be taken into account. This retrospective real-world study investigates the use of baloxavir for treating influenza in individuals aged 5 and above. In practical application, oseltamivir and baloxavir are utilized for antiviral treatment, and non-steroidal antipyretic and analgesic drugs are employed for symptomatic treatment to achieve swift fever reduction. While we made efforts to gather extensive data on the duration until patients completely ceased to have a fever following the administration of oseltamivir or baloxavir, there were no stringent constraints and records. Consequently, the time for complete fever reduction may still have been affected by the use of antipyretic and analgesic drugs, potentially leading to misjudgments by patients’ parents and resulting in a shorter perceived duration for complete fever reduction. However, this was an inevitable scenario in real-world research, and to a certain degree, the outcome may reflect the real-world situation.

Nevertheless, the constraints of research conditions and situations may introduce bias into our findings. Baloxavir was exclusively authorized for use in China as of March 2023, specifically for children aged 5 and above diagnosed with influenza. Thus, the administration of baloxavir for influenza treatment in children in China is at an initial phase. The study has gathered scant data on baloxavir, with a significantly lower number of children being treated with baloxavir compared to oseltamivir in clinical settings. This disparity may introduce bias into the research outcomes, potentially masking certain risks, particularly drug resistance. Previous research has established a correlation between the rise of baloxavir resistance and prolonged, extensive usage. The development of baloxavir resistance tends to manifest gradually as prescriptions increase over time (30, 31). Additionally, given the long half-life of baloxavir, only a single dose is required in clinical treatment. Therefore, the development of resistance to baloxavir is more likely, as resistance can emerge even after a single administration. This resistance may increase with prolonged and widespread use. Baloxavir was initially approved for the treatment of influenza in Japan. After widespread use during the influenza season, resistance of the influenza virus to baloxavir has rapidly increased, particularly among children (28, 32). Our research data originates from the preliminary phase of evaluating baloxavir for treating influenza in pediatric patients in China. Before this time, the drug had limited usage, resulting in minimal drug resistance and demonstrating favorable efficacy and sensitivity in treating influenza in children. The increasing prevalence of baloxavir utilization in Chinese children may lead to a gradual rise in drug resistance, posing new challenges in the management of influenza in this population. At present, the study does not elucidate the potential development of drug resistance to baloxavir with extended use. Long-term evaluation and further investigation in subsequent studies are essential.

Combination therapy has shown promising results in enhancing the treatment efficacy and reducing resistance in influenza infections. Various studies indicate that the synergistic effects of combining antiviral drugs can lead to improved outcomes and help mitigate the development of resistant viral strains. For instance, the combination of neuraminidase inhibitors and nitazoxanide has demonstrated a synergistic antiviral effect against influenza A viruses *in vitro*, attributable to their pharmacological actions rather than increased cytotoxicity (33). Similarly, the use of baloxavir and oseltamivir in mouse models has been effective in inhibiting the emergence of resistant substitutions in the influenza A virus PA gene, providing insights into alternative therapies to reduce antiviral resistance (34). Furthermore, targeting both viral and host factors has emerged as a novel approach. The combination of oseltamivir and itraconazole, an antifungal drug, significantly enhanced antiviral activity in polarized bronchial epithelial cells infected with influenza virus strains, suggesting that such combinations could protect against oseltamivir-resistant strains (35). Another study highlighted the potential of combining the MEK inhibitor ATR-002 with baloxavir, which showed synergistic efficacy against various influenza A virus strains, including those resistant to baloxavir (36). Additionally, the combination of baloxavir with other approved inhibitors like oseltamivir or favipiravir has been shown to have a synergistic effect on cell viability against different subtypes of influenza A viruses *in vitro*, further supporting the efficacy of combination therapy (37). In ferret models, combining baloxavir with oseltamivir reduced the selection of drug-resistant viral strains compared to monotherapy, indicating that such combinations could be more effective in clinical settings (38). Overall, the integration of host-directed therapies with traditional antiviral drugs represents a strategic approach to enhancing antiviral effects and overcoming resistance. By simultaneously targeting multiple pathways, combination therapies can achieve therapeutic success at lower doses and reduce the likelihood of resistance development, making them a critical component of influenza treatment strategies (39).

There were specific limitations: (1) This is a non-blind retrospective study. (2) In China, children attended outpatient clinics for a follow-up to obtain a diagnosis or a recovery certificate before returning to school, thus our second sampling coincided with this consultation. However, we could not control for other influencing factors such as underlying health conditions or medication compliance. (3) The symptom severity may not have been fully captured, as only the presence or absence was recorded, which hindered the observation of prominent differences between groups. Despite these limitations, the study provides valuable insights into the effectiveness of baloxavir and oseltamivir in treating influenza and highlights the need for further research in this area. This evidence is crucial in informing healthcare professionals and policymakers in their decision-making regarding the use of these antiviral medications in the treatment of influenza in children.

In conclusion, this study provides real-world evidence supporting the safety and efficacy of baloxavir and oseltamivir in the treatment of influenza among pediatric patients in China. The incidence of nausea and vomiting is lower with baloxavir compared to oseltamivir, and the duration of fever is shorter with baloxavir than with oseltamivir. The results indicate that baloxavir is well tolerated and effective.

## Data Availability

The original contributions presented in the study are included in the article/Supplementary Material, further inquiries can be directed to the corresponding authors.
